# Fruit intake, genetic risk and type 2 diabetes: a population-based gene–diet interaction analysis

**DOI:** 10.1007/s00394-020-02449-0

**Published:** 2021-01-05

**Authors:** Xu Jia, Liping Xuan, Huajie Dai, Wen Zhu, Chanjuan Deng, Tiange Wang, Mian Li, Zhiyun Zhao, Yu Xu, Jieli Lu, Yufang Bi, Weiqing Wang, Yuhong Chen, Min Xu, Guang Ning

**Affiliations:** 1 Department of Endocrine and Metabolic Diseases, Shanghai Institute of Endocrine and Metabolic Diseases, Ruijin Hospital, Shanghai Jiao Tong University School of Medicine, 197 Ruijin 2nd Road, Shanghai, 200025 China; 2 Shanghai National Clinical Research Center for Metabolic Diseases, Key Laboratory for Endocrine and Metabolic Diseases of the National Health Commission of the PR China, Shanghai National Center for Translational Medicine, Ruijin Hospital, Shanghai Jiao Tong University School of Medicine, Shanghai, China

**Keywords:** Type 2 diabetes, Fruit intake, Gene–diet interaction, Genetic risk score, Glycemic traits

## Abstract

**Purpose:**

Whether the association between fruit and type 2 diabetes (T2D) is modified by the genetic predisposition of T2D was yet elucidated. The current study is meant to examine the gene–dietary fruit intake interactions in the risk of T2D and related glycemic traits.

**Methods:**

We performed a cross-sectional study in 11,657 participants aged ≥ 40 years from a community-based population in Shanghai, China. Fruit intake information was collected by a validated food frequency questionnaire by asking the frequency of consumption of typical food items over the previous 12 months. T2D-genetic risk score (GRS) was constructed by 34 well established T2D common variants in East Asians. The risk of T2D, fasting, 2 h-postprandial plasma glucose, and glycated hemoglobin A1c associated with T2D-GRS and each individual single nucleotide polymorphisms (SNPs) were tested.

**Results:**

The risk of T2D associated with each 1-point of T2D-GRS was gradually decreased from the lower fruit intake level (< 1 times/week) [the odds ratio (OR) and 95% confidence interval (CI) was 1.10 (1.07–1.13)], to higher levels (1–3 and > 3 times/week) [the corresponding ORs and 95% CIs were 1.08 (1.05–1.10) and 1.07 (1.05–1.08); *P* for interaction = 0.04]. Analyses for associations with fasting, 2 h-postprandial plasma glucose and glycated hemoglobin A1c demonstrated consistent tendencies (all *P* for interaction ≤ 0.03). The inverse associations of fruit intake with risk of T2D and glucose traits were more prominent in the higher T2D-GRS tertile.

**Conclusions:**

Fruit intakes interact with the genetic predisposition of T2D on the risk of diabetes and related glucose metabolic traits. Fruit intake alleviates the association between genetic predisposition of T2D and the risk of diabetes; the association of fruit intake with a lower risk of diabetes was more prominent in population with a stronger genetic predisposition of T2D.

**Supplementary Information:**

The online version contains supplementary material available at 10.1007/s00394-020-02449-0.

## Introduction

Diabetes has become a worldwide epidemic [1]. As a complex disease triggered by multiple factors, hereditary predispositions and unhealthy diet are believed the two major etiological incentives. In the past decade, hundreds of genetic loci for type 2 diabetes (T2D), obesity, and other metabolic traits were identified due to the rapid development of genotyping and sequencing techniques that vigorously promoted understandings the genetic architecture of metabolic diseases [2, 3]. By taking advantage of this opportunity, the interactive effect between dietary and genetic factors began to highlight recent studies [4–9]. These findings emphasized that active interactions exist between dietary factors and genetic predispositions. Specifically, individuals who adopted different dietary patterns may present disparate risks of diseases even if comparable genetic susceptibility were shared. In the same way, people having similar dietary habits can display dramatic phenotypical differences because of distinct genetic predispositions [10]. Thus, investigations for potential gene–diet interactions could be essential to explore novel plans of personalized management and promote precision medicine for chronic diseases.

Fruits are a set of nutritious plant products renowned for the various phytochemicals, rich vitamins and minerals, while low energy density contained. Taking fruits more frequently has been proved beneficial in reducing risks of multiple chronic diseases including T2D, stroke, and cardiovascular diseases [11–15, 16]. Therefore, we hypothesized a gene–diet interaction with regard to the risk of T2D between fruit intake and T2D genes. To test this hypothesis, we investigated the associations of fresh fruit intakes, a genetic risk score (GRS) consisted of 34 common variants well established to be associated with T2D in East Asians, with the risk of the presence of T2D and related glucose metabolic traits in a Chinese community-based population; we particularly investigated the interactions of fresh fruit intakes and GRS in these associations.

## Materials and methods

### Study population

As a cross-sectional study of gene–diet interaction, our study was based on part of a nationwide survey of the Risk Evaluation of cAncers in Chinese diabeTic Individuals: a lONgitudinal (REACTION) study, which is a large cohort involving 259,657 community-based population, aged 40 years or older [17–20]. In brief, all the participants were recruited from two nearby communities in Baoshan district of Shanghai, China, during 2011 and 2013.

There were 11,935 participants recruited in the study. Food frequency information was available in 11,884 participants (99.6%). Among which, subjects with more than two single nucleotide polymorphisms (SNPs) genotype information missed were excluded (*n* = 227) and 11,657 (97.7%) participants were finally involved in the current study. The flow chart for participant recruitment was shown in Supplemental Fig. 1.

The Institutional Review Board of Rui-Jin Hospital, Shanghai Jiao Tong University School of Medicine, approved the study protocol. Each participant gave the written informed consent.

### Genotyping and quality control

Blood white cells were collected for DNA extractions using commercial blood genomic DNA extraction kit (OSR-M102-T1, TIANGEN BIOTECH CO, LTD, Beijing, China). The minimum call rate was 98.7%. The concordance rate is more than 99% based on 100 duplicates genotyping.

### Genetic loci selection and GRS construction

The selection and GRS creation methods were extensively described in our previous papers [19–21]. On considering the population specificity of genetic background, the selected SNPs were discovered or replicated by genome-wide association studies (GWASs) in East Asians [22–24]. For the GRS construction, we assumed the additive genetic model by applying a linear weighting of 0, 1, and 2 to genotypes containing 0, 1, or 2 risk alleles for each SNP [25]. The weighted GRS was the sum of the number of risk alleles weighted by the effect for the risk of T2D summarized in the literature. Using these 34 SNPs, we constructed a weighted GRS (mean ± SD, 34.56 ± 3.89) for main analyses and an un-weighted GRS (mean ± SD, 35.88 ± 3.61) for sensitivity analyses.

### Assessment of fruit intake frequency

To minimize the bias, dietary habits were collected by well-trained interviewers with a validated semi-quantitative food frequency questionnaire (FFQ) comprised of standardized questions [26]. Specifically, the FFQ inquired for consumption frequencies of 21 major food categories which were often consumed in the Chinese population, including grains, tubers, fresh fruits, vegetables, eggs, aquatic products, pork, beef and mutton, poultries, offal, bean products, dairy products, fried food, cake and pastry, freshly squeezed juices, fruit-flavored beverages, carbonated beverages, coffee, pickled vegetables, fermented bean curd, and dietary supplements. Subjects were asked to estimate the number of times that they habitually consumed daily, weekly, monthly or annually (choose one from the four to fill in) for each interested food in the last 12 months; all the responses were converted to daily frequency later. In the current study, the fruit intake frequencies were coded into three levels, namely, “less than once per week” (< 1 times/week), “1–3 times per week” (1–3 times/week), and “more than 3 times per week” (> 3 times/week).

### Definition of diabetes

Fasting plasma glucose (FPG) and 2-h plasma glucose (2 h-PG) were measured using the hexokinase method on a clinical chemistry diagnostic system (C16000, Abbott Laboratories, Illinois, USA). According to the American Diabetes Association 2010 criteria, T2D was defined as FPG ≥ 7.0 mmol/L, 2 h-PG ≥ 11.1 mmol/L or glycated hemoglobin A1c (HbA1c) ≥ 6.5% or self-reported previous physician-diagnosed diabetes and use of anti-diabetic agents.

### Assessment of covariates

We used a standard questionnaire to collect lifestyle factors including habits of smoking, drinking and physical activity, etc. The current smoking or drinking status was defined as “yes” if the subject smoked at least one cigarette or consumed alcohol at least once a week in the past 6 months. Physical activity at leisure time was assessed using the short form of the International Physical Activity Questionnaire [27] by adding questions on the duration of mild/moderate/vigorous activities per day. Body mass index (BMI) was calculated as body weight in kilograms divided by height squared in meters (kg/m^2^). Systolic and diastolic blood pressure (SBP and DBP) were measured in triplicate on the same day after at least 10-min rest using an automated electronic device (OMRON Model HEM-752 FUZZY, Omron Company, Dalian, China).

Fasting serum triglycerides (TG), total cholesterol (TC), low-density lipoprotein cholesterol (LDL-C) and high-density lipoprotein cholesterol (HDL-C) were measured by the clinical chemistry diagnostic system (C16000, Abbott Laboratories, Illinois, USA). Plasma glycated hemoglobin A1c (HbA1c) was measured by high-performance liquid chromatography using the VARIANT II Hemoglobin Testing System (Bio-Rad Laboratories).

### Statistical analysis

The homeostasis model assessment for β cell function (HOMA-β) was calculated with the formula “20 × fasting insulin (mIU/L)/[FPG (mmol/L) − 3.5]”; the homeostasis model assessment for insulin resistance (HOMA-IR) was calculated with the formula the “fasting insulin (mIU/L) × FPG (mmol/L)/22.5”. The principal component analysis was adopted to extract effective information on dietary factors other than fruit intake.

We first test the main effect of fruit intake level and T2D-GRS with the risk of T2D, respectively, using multiple logistic regression analyses; age, gender, and BMI were adjusted as Model 1, while SBP, DBP, Log-TG, TC, LDL-C, HDL-C, smoking and drinking status, physical activity and principal components of dietary factors were further adjusted as Model 2. We particularly tested the interaction effect of fruit intake with T2D-GRS on the risk of T2D by multiple logistic regression analysis under both Model 1 and Model 2, in which T2D-GRS, fruit intake level, T2D-GRS × fruit intake level term, and covariates were together taken as independent variables. Then we performed the stratified analysis to examine the association of T2D-GRS with the risk of T2D in each fruit intake level; and inversely, the association of fruit intake level with the risk of T2D in each tertile of T2D-GRS.

Subsequently, to test the possibility of reverse causation, we performed a sensitivity analysis by repeated the above procedures in two subpopulations with no self-awareness of T2D (*N* = 9946) or no dietary interventions for T2D (*N* = 10,105), respectively.

We also tested the interactions of fruit intake with T2D-GRS on FPG, 2 h-PG, HbA1c, Log-HOMA-β, and Log-HOMA-IR by multiple linear regression analyses. In addition to the Model 1 and Model2, we further adjusted self-awareness of diabetes, diet and exercise intervention for diabetes, and diabetic treatment to eliminate the potential influence of lifestyle change after a diagnosis of diabetes and antidiabetic agents as Model 3.

To detect the interactions for individual SNPs, we introduced the 2-degree of freedom (df) test which was developed to detect the main genetic effects and the gene–environment interactions simultaneously [28]. Specifically, likelihood ratio tests between the full linear regression model (SNP, fruit intake levels, fruit intake levels × SNP, and covariates) and the reduced model (fruit intake levels and covariates only) were performed; *P* values of the Chi-square were calculated using the differences between the *-2LogL(*β*)*. Then, the conventional interaction test was performed again for SNPs passed the significance threshold of the previous joint tests. Finally, SNPs with the *P* value less than the value after Bonferroni correction (*P* < 0.002) were considered significant [29].

Analyses in the current study were performed by SAS version 9.4 (SAS Institute, Cary, NC). All tests were two-tailed and a *P* value < 0.05 was considered statistically significant.

## Results

Of the 11,657 participants, 4150 (35%) were men, the age ranged from 31 to 93 years (mean, 63.3 years; median, 62.0 years), and the average BMI was 25.24 kg/m^2^ [standard deviation (SD), 3.51]. Subjects with diabetes accounted for 27% of participants, and the weighted T2D-GRS ranged from 21.02 to 49.39 with an average of 34.56. As for the fruit intake levels, 2061 (18%) participants were in the first level (< 1 times/week), 3075 (26%) in the second (1–3/week), and 6521 (56%) in the third (> 3 times/week).

The demographic and biochemical characteristic of the study participants was presented in Supplemental Table 2. The T2D-GRS, BMI, SBP, DBP, FPG, 2 h-PG, and log-HOMA-IR were significantly lower in the higher level of fruit intake group, while the HDL-C and Log-HOMA-β were higher along with the higher level of fruit intake (all *P* < 0.01). Women took fruit more frequently than men (*P* < 0.0001). In addition, in the higher frequency fruit intake group, the diabetes prevalence was significantly declined.

Multiple logistic regression analyses showed that, each 1-point increase in T2D-GRS was associated with 8.0% [odds ration (OR), 1.08; 95% confidence interval (CI), 1.07–1.09] increment of the risk of diabetes in Model 1, and the results were the same in Model 2. And each 1-level higher fruit intake, namely, every one categorical increment we defined before, was associated with a 31% (OR 0.69; CI 0.65–0.73) lower of diabetes risk in Model 1, and 36% (OR 0.64; CI 0.60–0.68) in Model 2.

**Fig. 1:**
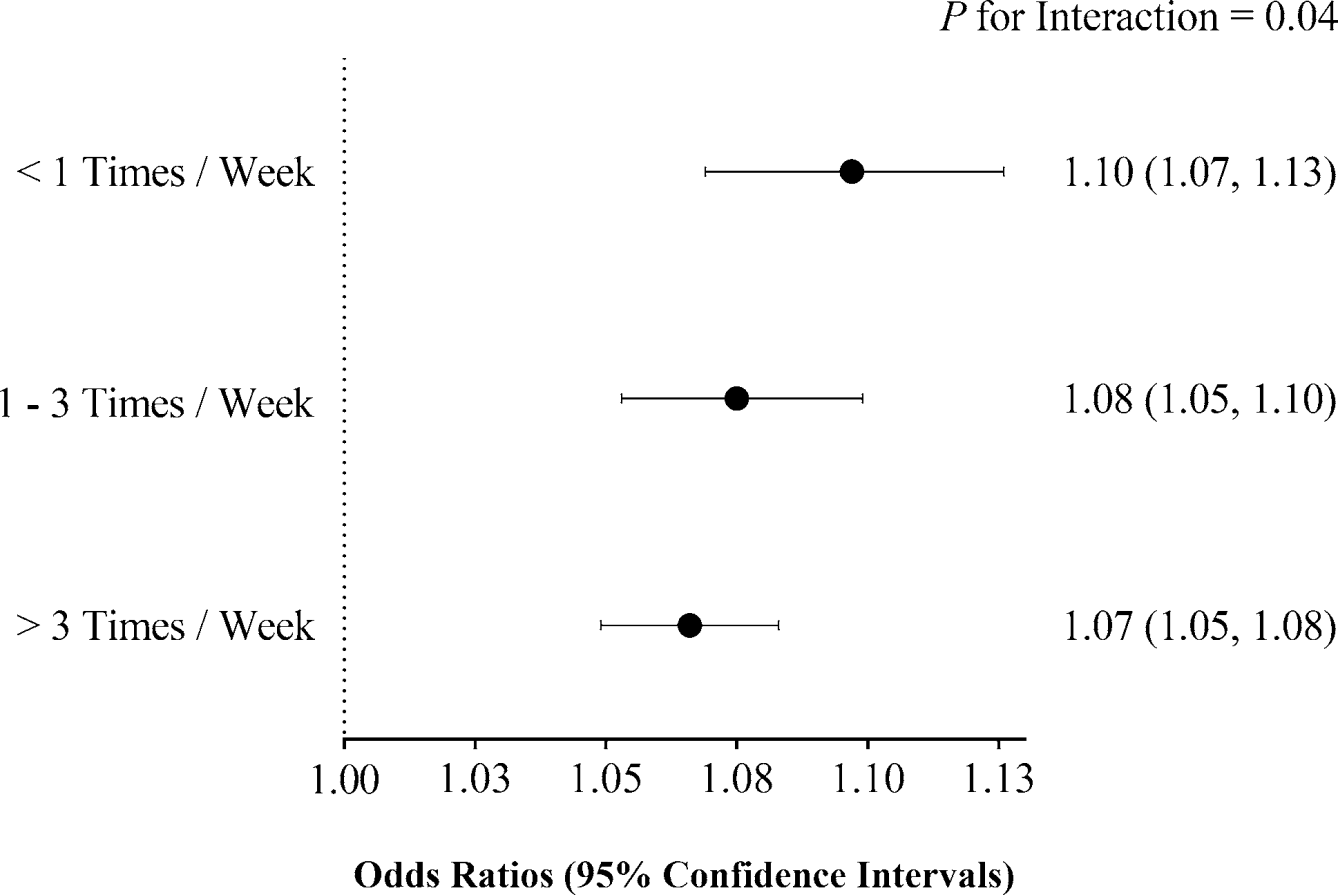
Association of T2D-GRS with risk of T2D stratified by different fruit intake levels. The odds ratios [OR, 95% confidence intervals (CI)] under each fruit intake levels were derived from multiple logistic regression models using the T2D-GRS and covariates as independent variables; the *P* for interaction values were calculated using the T2D-GRS, fruit intake level, T2D-GRS × fruit intake level, and covariates together as independent variables. The shown data were after adjustments for age, gender, BMI, SBP, DBP, Log-TG, TC, LDL-C, HDL-C, smoking, drinking, physical activity and principle components of dietary factors

We detected a significant T2D-GRS and fruit intake interaction in the risk of T2D (*P* for interaction = 0.03 in Model 1 and 0.04 in Model 2). The ORs of diabetes for each 1-point increase in T2D-GRS were attenuating progressively as the fruit intake levels ascended. Specifically, in Model 1, the OR of diabetes was 1.10 (CI 1.07–1.13) in the first fruit intake level (< 1 times/week), 1.07 (CI 1.05–1.10) in the second level (1–3/week), and 1.07 (CI 1.05–1.08) in the third level (> 3 times/week); in Model 2, the corresponding value was 1.10 (CI 1.07–1.13), 1.08 (CI 1.05–1.10), and 1.07 (CI 1.05–1.08) (Fig. 1).

Table 1 shows the association of fruit intake with the risk of diabetes stratified by T2D-GRS tertiles. Higher fruit intake was significantly associated with a lower risk of T2D; and such effect was increasingly sharper as the tertile ascended (*P* for interaction ≤ 0.04). In the full adjusted model (Model 2), for participants in the lowest tertile of T2D-GRS, > 3 times/week fruit intake was associated with about 56% lower risk of T2D (OR = 0.44, 95% CI 0.35, 0.56) as compared to < 1 times/week (*P* < 0.0001); the corresponding ORs were 0.40 (0.33–0.50) in participants at tertile 2 of T2D-GRS and decreased to 0.34 (0.27–0.41) in those at tertile 3 of T2D-GRS (both *P* < 0.0001) (*P* for interaction = 0.04).

**Table 1:** The risk of T2D presence associated with fruit intake stratified by T2D-GRS tertiles

	Fruit intake Levels	T2D-GRS						*P* for Interaction
		Tertile 1 (*n* = 3885)		Tertile 2 (*n* = 3885)		Tertile 3 (*n* = 3887)		
		OR (95% CI)	*P* value	OR (95% CI)	*P* value	OR (95% CI)	*P* value	
Model 1	< 1 times/week	1.00		1.00		1.00		0.03*
	1–3 times/week	0.69 (0.55, 0.86)	0.61	0.58 (0.47, 0.72)	0.03*	0.57 (0.46, 0.69)	0.15	
	> 3 times/week	0.52 (0.42, 0.64)	< 0.0001^†^	0.49 (0.41, 0.59)	< 0.0001^†^	0.41 (0.34, 0.49)	< 0.0001^†^	
Model 2	< 1 times/week	1.00		1.00		1.00		0.04*
	1–3 times/week	0.60 (0.46, 0.76)	0.26	0.48 (0.38, 0.60)	0.003^†^	0.49 (0.40, 0.61)	0.06	
	> 3 times/week	0.44 (0.35, 0.56)	< 0.0001^†^	0.40 (0.33, 0.50)	< 0.0001^†^	0.34 (0.27, 0.41)	< 0.0001^†^	

Data are odds ratios (ORs) and 95% confidence intervals (CI). *P* values under each T2D-GRS tertiles were derived from multiple logistic regressions using the fruit intake level and covariates as independent variables. *P* for interaction values were calculated using the T2D-GRS tertile, fruit intake level, T2D-GRS × fruit intake level, and covariates together as independent variables. Model 1 adjusted for age, gender, and body mass index; Model 2 additionally adjusted for systolic and diastolic blood pressure, log transformed triglyceride, total cholesterol, low- and high-density lipoprotein cholesterol, smoking, drinking, physical activity and principal components of dietary factors. *P < 0.05; ^†^*P* < 0.01

Although the *P* for interaction of the sensitivity analyses using subpopulations with no self-awareness of T2D (*P* = 0.34) or no dietary interventions for T2D (*P* = 0.10) failed to pass the significant threshold given the loss of sample size, the trends of variation from the lower to the higher strata were in highly consistent with the analysis outcome in the integral population (Supplemental Tables 3, 4).

**Fig. 2:**
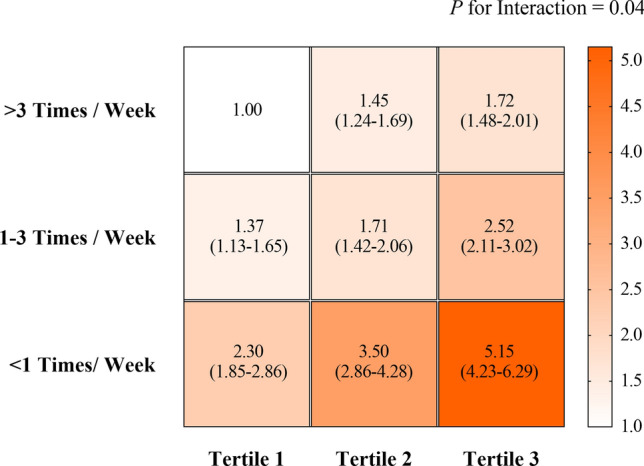
The joint effect of fruit intake levels and T2D-GRS on risk of T2D. The participants who were with the highest T2D-GRS tertile and the lowest level of fruit intake (< 1 times/week) were taken as the reference. Data in each square are odds ratios [ORs, 95% confidence interval (CI)] of T2D. The right bar with color gradient was presented the range of OR values. The *P* for interaction values were calculated using the T2D-GRS, fruit intake level, T2D-GRS × fruit intake level, and covariates together as independent variables. All tests were performed after adjustments for age, gender, BMI, SBP, DBP, Log-TG, TC, LDL-C, HDL-C, smoking, drinking, physical activity and principal components of dietary factors

By taking participants in the first T2D-GRS tertile with the highest fruit intake level as a reference, we further tested the risk of diabetes for combination of each tertile of T2D-GRS and fruit intake level. In general, the risk of diabetes presence gradually increased in lower fruit intake level and higher of T2D-GRS tertile, and it was maximized for those with both fruit intake level of < 1 times/week and highest tertile of T2D-GRS (OR 5.15, 95% CI 4.23–6.29) (Fig. 2).

**Fig. 3:**
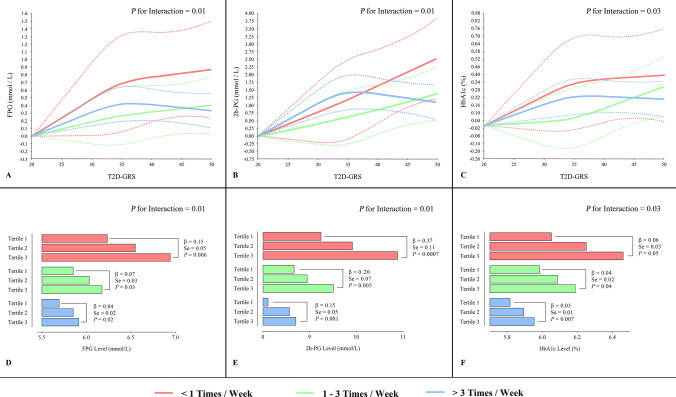
Spline analysis for association of T2D-GRS and tertiles of T2D-GRS with FPG, 2 h-PG, and HbA1c stratified by fruit intake levels. **a**–**c** Association of T2D-GRS with FPG, 2 h-PG, and HbA1c stratified by fruit intake levels. Solid curves were stringent cubic splines representing the linear associations; dot lines were corresponding 95% confidence intervals. *P* for interaction were calculated with the T2D-GRS, fruit intake level, T2D-GRS × fruit intake level, and covariates together as independent variables. **d**–**e** Association of T2D-GRS tertile with FPG, 2 h-PG, and HbA1c stratified by fruit intake levels. β coefficients, SE and *P* values were derived from multiple linear regressions with T2D-GRS tertile and covariates as independent variables; *P* for interaction were calculated with the T2D-GRS tertile, fruit intake level, T2D-GRS tertile fruit intake level, and covariates together as independent variables. All tests were after adjustments for age, gender, BMI, SBP, DBP, Log-TG, TC, LDL-C, HDL-C, smoking, drinking, physical activity, principle components of dietary factors, self-awareness of diabetes, exercise and diet intervention, and diabetic treatment

Table 2 shows the association of T2D-GRS with the glucose metabolism related traits stratified by fruit intake levels. In Model 1 and 2, the results for association with FPG, 2 h-PG and HbA1c demonstrated similar tendencies as those with diabetes. The effect size of the association of GRS with T2D was decreased along with increase of fruit intake; all *P* for interaction < 0.0001. Given the potential influence of antidiabetic agents and lifestyle changes after diagnosis of T2D, we further adjusted self-awareness of diabetes, diet and exercise intervention for diabetes, and diabetic treatment in the Model 3, the results did not appreciably change (all *P* for interaction ≤ 0.03) (Table 2). The cubic spline analysis of association of GRS with FBG, 2 h-PG and A1c (Fig. 3a–c) and linear association of tertiles of GRS (Fig. 3d–f) with these metabolic traits by 3 fruit intake levels confirmed the above findings.

**Table 2:** The association of each 1-point T2D-GRS with FPG, 2 h-PG, Log-HOMA-β, and Log-HOMA-IR stratified by fruit intake levels

	< 1 times/week		1–3 times/week		> 3 times/week		*P* for interaction
	β ± SE	*P* value	β ± SE	*P* value	β ± SE	*P* value	
FPG, mmol/L							
Model 1	0.08 ± 0.01	< 0.0001^†^	0.04 ± 0.008	< 0.0001^†^	0.03 ± 0.005	< 0.0001^†^	< 0.0001^†^
Model 2	0.08 ± 0.01	< 0.0001^†^	0.04 ± 0.008	< 0.0001^†^	0.03 ± 0.004	< 0.0001^†^	< 0.0001^†^
Model 3	0.03 ± 0.01	0.007^†^	0.01 ± 0.006	0.04*	0.01 ± 0.004	0.002^†^	0.01*
2 h-PG, mmol/L							
Model 1	0.20 ± 0.03	< 0.0001^†^	0.10 ± 0.02	< 0.0001^†^	0.08 ± 0.01	< 0.0001^†^	< 0.0001^†^
Model 2	0.19 ± 0.03	< 0.0001^†^	0.10 ± 0.02	< 0.0001^†^	0.07 ± 0.01	< 0.0001^†^	< 0.0001^†^
Model 3	0.08 ± 0.02	0.0002^†^	0.05 ± 0.02	0.002†	0.04 ± 0.01	< 0.0001^†^	0.01*
HbA1c, %							
Model 1	0.05 ± 0.008	< 0.0001^†^	0.03 ± 0.005	< 0.0001^†^	0.02 ± 0.003	< 0.0001^†^	< 0.0001^†^
Model 2	0.04 ± 0.008	< 0.0001^†^	0.02 ± 0.005	< 0.0001^†^	0.02 ± 0.003	< 0.0001^†^	< 0.0001^†^
Model 3	0.01 ± 0.006	0.03*	0.01 ± 0.004	0.01*	0.007 ± 0.002	0.002†	0.03*
Log-HOMA-β							
Model 1	− 0.03 ± 0.004	< 0.0001^†^	− 0.01 ± 0.003	< 0.0001^†^	− 0.02 ± 0.002	< 0.0001^†^	0.005^†^
Model 2	− 0.03 ± 0.004	< 0.0001^†^	− 0.01 ± 0.003	< 0.0001^†^	− 0.01 ± 0.002	< 0.0001^†^	0.004^†^
Model 3	− 0.02 ± 0.003	< 0.0001^†^	− 0.007 ± 0.002	0.003^†^	− 0.01 ± 0.002	< 0.0001^†^	0.24
Log-HOMA-IR							
Model 1	0.006 ± 0.003	0.04*	0.006 ± 0.002	0.009^†^	0.0001 ± 0.001	0.93	0.02*
Model 2	0.005 ± 0.003	0.05	0.005 ± 0.002	0.01*	− 0.00008 ± 0.001	0.95	0.01*
Model 3	− 0.0009 ± 0.003	0.74	0.002 ± 0.002	0.25	− 0.002 ± 0.001	0.12	0.21

Data are β-coefficients ± standard error (SE). *P* values under each fruit intake levels were derived from multiple linear regression models using the T2D-GRS and covariates as independent variables, while the *P* for interaction were calculated using the T2D-GRS, fruit intake level, T2D-GRS × fruit intake level, and covariates together as independent variables. Model 1 adjusted for age, gender, and body mass index; Model 2 additionally adjusted for systolic and diastolic blood pressure, log transformed triglyceride, total cholesterol, low- and high-density lipoprotein cholesterol, smoking, drinking, physical activity, and principal components of dietary factors; Model 3 further adjusted for self-awareness of diabetes, exercise and diet intervention, and diabetic treatment. FPG, fasting plasma glucose; 2 h-PG, OGTT 2-h plasma glucose; HbA1c, glycated hemoglobin A1c; Log-HOMA-β, log transformed homeostasis model assessment for β cell function; Log-HOMA-IR, log transformed homeostasis model assessment for insulin resistance. *P < 0.05; ^†^*P* < 0.01

We also analyzed the interaction of GRS and fruit intake in influencing Log-HOMA-β and Log-HOMA-IR level (Table 2). After the full adjustments (Model 3), we did not find a significant interaction effect of GRS and fruit intake, though in each stratum of fruit intake level, the GRS was significantly associated with a lower HOMA-β level (both *P* ≤ 0.003**).**

The association of fruit intake level with FPG, 2 h-PG, HbA1c, Log-HOMA-β, and Log-HOMA-IR stratified by the T2D-GRS tertiles was displayed in Supplemental Table 5. Briefly, in Model 3, the association of fruit intake with FPG, 2 h-PG and HbA1c were more prominent in the higher tertile of GRS (all *P* for interaction ≤ 0.05). No significant interactions for Log-HOMA-β and Log-HOMA-IR.

Table 3 shows the results of interaction analyses for each individual SNP on the risk of diabetes. 24 SNPs that passed the screen (*P* < 0.05) of the 2-df joint test were further tested by the conventional 1-df method. Of which, 2 SNPs, namely, rs10906115 at *CDC123/CAMK1D* and rs7172432 at *C2CD4B/C2CD4A*, passed the threshold of *P* < 0.05, while only rs10906115 (*P* for interaction = 0.001) remained significant after the Bonferroni correction. Therefore, we further tested the interactive effect of rs10906115 at *CDC123/CAMK1D* and rs7172432 at *C2CD4B/C2CD4A* with fruit intake level on FPG, 2 h-PG, HbA1c, Log-HOMA-β, and Log-HOMA-IR with full adjustments (Model 3). The interaction of both the two variants remained significant in analyses for FPG, 2 h-PG, HbA1c (all *P* for Interaction < 0.05) (Supplemental Table 6).

**Table 3:** Individual SNPs passed the screening of the 2-df joint test and the outcomes of the corresponding conventional 1-df test

SNPs	Genes	Identification population	− 2Log* L* (*β*) difference	*P* for 2-df screen	*P* for 1-df test
rs10906115	*CDC123/CAMK1D*	East Asian	21.05	< 0.0001^†^	0.001^**‡**^
rs7172432	*C2CD4B/NPM1P47*	East Asian	6.37	0.02*	0.03*
rs35612982	*CDKAL1*	East Asian	52.14	< 0.0001^†^	0.05
rs1801282	*PPARG*	European	6.32	0.02*	0.06
rs340874	*PROX1*	Multi-ethnic	7.77	0.01*	0.07
rs9936385	*FTO*	Multi-ethnic	6.47	0.02*	0.12
rs13266634	*SLC30A8*	European	38.92	< 0.0001^†^	0.13
rs7612463	*UBE2E2*	Multi-ethnic	14.95	0.0003^†^	0.14
rs6815464	*MAEA*	East Asian	4.62	0.05*	0.17
rs780094	*GCKR*	European	5.44	0.03*	0.21
rs231362	*KCNQ1*	European	5.82	0.03*	0.21
rs10811661	*CDKN2A/B*	European	26.75	< 0.0001^†^	0.22
rs1552224	*CENTD2*	European	4.96	0.04*	0.25
rs243021	*BCL11*	European	9.41	0.005^†^	0.38
rs2237892	*KCNQ1*	East Asian	38.84	< 0.0001^†^	0.40
rs2191349	*DGKB*	Multi-ethnic	30.72	< 0.0001^†^	0.44
rs5215	*KCNJ11*	European	5.92	0.03*	0.69
rs4430796	*TCF2/HNF1B*	European	23.99	< 0.0001^†^	0.71
rs4402960	*IGF2BP2*	European	17.02	0.0001^†^	0.72
rs896854	*TP53INP1*	European	7.65	0.01*	0.76
rs2943641	*IRS1*	European	14.45	0.0004^†^	0.87
rs1359790	*SPRY2*	East Asian	12.06	0.001^†^	0.89
rs1111875	*HHEX/IDE*	European	10.44	0.003^†^	0.89
rs7903146	*TCF7L2*	European	6.39	0.02*	0.96

The *P* for 2-df screen were calculated by Chi-square test using difference between *-2LogL(*β*)* derived from the full multiple logistic regression model with SNP, fruit intake level, SNP × fruit intake level, and covariates as independent variables and the reduced model with only fruit intake level and covariates. The *P* for 1-df test were derived from multiple logistic regression model with SNP, fruit intake level, SNP × fruit intake level, and covariates as independent variables. All tests were under the Model 2, which adjusted for age, gender, body mass index, systolic and diastolic blood pressure, log transformed triglyceride, total cholesterol, low- and high-density lipoprotein cholesterol, smoking, drinking, physical activity, and principal components of dietary factors. *df* degree of freedom, *SNP* single nucleotide polymorphism. *P < 0.05; ^†^*P* < 0.01; ^**‡**^*P* < 0.002 (≈ 0.05/24)

## Discussion

According to the results of the present study, fruit intake significantly modified the genetic association with T2D, FPG, 2 h-PG and HbA1c. The association of GRS with T2D and glucose traits were attenuated in a higher level of fruit intake. Meanwhile, the inverse associations of fruit intake with T2D and glucose traits were more prominent in the higher GRS groups. Among the 34 common variants adopted in the construction of the GRS, 24 were identified significant in the screening tests for interactions of individual SNP, of which rs10906115 at *CDC123/CAMK1D* passed the significance threshold after Bonferroni correction.

To date, the relationship between fruit intake and the risk of diabetes has been widely studied. Despite of mixed outcomes [30, 31] and concerns on glucose-load burdened by fruits with high glycemic index [32], the majority of studies tended to support the beneficial role of fruits in lowering the risk of diabetes. In particular, a longitudinal study combined 3 cohorts with 3,464,641 person-year of follow-up in total proved that higher consumption of blueberries, grapes, and apples was significantly associated with lower risks of T2D [11]. A recent study on a large Chinese cohort demonstrated that higher fresh fruit intake significantly reduced the risk of diabetes [13]. Noteworthily, a most recent cohort study in a Chinese population demonstrated that the protective effect against T2D of a healthy lifestyle characterized by more daily fruit and less meat consumption was independent of genetic susceptibility of T2D [33]. However, the reciprocal effects among fruit, T2D, and its genetic predisposition were yet well elucidated [34]. In line with the previous epidemiology studies, the results in the present study demonstrated that fruit intake was inversely associated with the risk of diabetes presence; in particular, our analyses provided novel evidence for the interaction of fruit intake with the genetic association of diabetes. Our study was in accordance with a previous gene–diet interaction analysis, in which western dietary pattern (relatively lower consumption of fresh fruits) was proven significantly associated with a stronger genetic association of T2D in men [35].

The onset and prognosis of diabetes depend on the regulation of both internal hereditary and external environmental factors. Actually, a number of studies identified interplays between T2D associated variants and dietary factors on the risk of diabetes. An early interaction analysis suggests that the intake of dietary fiber modified the association of rs7903146 at *TCF7L2* with T2D incidence [36]. Another case–cohort study also identified evidence for a possible interaction of *TCF7L2* variants with coffee consumption in relation to T2D risk [37]. The p. R270H variant at *FFAR4* and rs2943641 at *IRS1* was proven functional in modulating the association of dietary fat or carbohydrate intake with T2D risk [38, 39]. In the present study, SNPs at *TCF7L2* and *IRS1* also passed the screen of the 2-df joint test but failed in the further verification of the conventional 1-df test.

Interestingly, the only SNP passed the Bonferroni correction (rs10906115) and the other SNP that passed the *P* < 0.05 threshold (rs7172432) were all T2D associated common variants which discovered in Eastern Asian populations [40–42]. Such a result might suggest a possibly stronger interaction of fruit intake with genetically determined risk of T2D particularly in Eastern Asian populations.

Mechanism studies inferred that fruits and the phytochemicals contained may play an important role in DNA methylation [43, 44]. Vitamins were also proven functional in modulating the risks of chronic conditions via gene expression or DNA methylations [45, 46]. In addition, nutrigenetic studies found that nutrients like polyphenols, flavan-3-ols, naringin, hesperidin and quercetin can positively affect genes involved in insulin synthesis, stimulus-secretion coupling, anti-glucolipotoxicity, inflammation, oxidative stress, and insulin resistance [47–51]] However, the exact mechanisms of the genetic effect on the predisposition of T2D under different fruit intake level were remained elucidated.

The strength of the current study was evidenced by a well-defined community setting, fair sized sample volume, desirable population homogeneity, and repeatedly validated information with regard to dietary and lifestyle factors. Meanwhile, we acknowledge the following limitations in our study. Firstly, the outcomes derived from the present study were based on a cross-sectional dataset, and therefore, the possibility of reverse causality could not be eliminated. Although multiple measures, including (1) performing sensitivity analysis for the odds of T2D presence by excluding diabetics with self-awareness of T2D or dietary intervention, and (2) adjusting self-awareness of T2D, diet and exercise intervention, and diabetic treatment in analyses for plasma glucose and HbA1c, prospective cohort studies are anticipated to confirm this interactive effect in regard with the incidence of diabetes. Secondly, the GRS integrated genetic effect from identified SNPs associated with T2D, whereas the loci involved only accounted for a minor portion of genetic predisposition of T2D [2]; however, we selected those genetic loci that were either identified in East Asians or identified in Europeans but successfully validated in East Asians through large genome wide association studies with robust association with T2D. Thirdly, the variants adopted in GRS construction were all common variants; the heritability of low frequency and rare variants could hardly be evaluated. Fourthly, although our study had multiple anthropometric, biochemical, and lifestyles corrected, residual confounding by other unmeasured or unknown factors might be omitted. Lastly, given the highly consistent composition of the population analyzed and some SNP used were validated only in Eastern Asians, it should be more cautious to generalize the results to other ethnic groups.

In conclusion, the present study provided evidence for interactions between fruit intakes and genetic predisposition of T2D with the risk of diabetes and related glucose metabolic traits in a Chinese community-based population. Dietary fresh fruit intakes alleviate the association of the T2D-GRS with the risk of diabetes and the increment in FPG, 2 h-PG and HbA1c levels. Also, the association between fresh fruit intake with a lower risk of diabetes and decrement of plasma glucose were more prominent in higher T2D-GRS. These results may throw light on the future gene–diet interaction studies for T2D, while validation of large prospective cohort study and effect explanations from mechanism researches are still anticipated before the conclusion been further applied to precision prevention and treatment of T2D.

## Supplementary Information

Below is the link to the electronic supplementary material.

Supplementary file1 (DOCX 123 KB)

## Data Availability

Data are available from the authors on request.
