# Supplementary material for: Fruit intake, genetic risk and type 2 diabetes: a population-based gene–diet interaction analysis
Source: Eur J Nutr. 2021 Jan 5;60(5):2769–79. doi: 10.1007/s00394-020-02449-0 (PMC8275558; doi:10.1007/s00394-020-02449-0)

**Online Supporting Material**

**Supplemental Table 1.** Information and effect of each individual SNP adopted.

**Supplemental Table 2.** Demographic information, metabolic traits, and lifestyles categorized by fruit intake levels.

**Supplemental Table 3.** The risk of T2D presence associated with T2D-GRS stratified by fruit intake in the subpopulations with no self-awareness of T2D or no dietary intervention for T2D.

**Supplemental Table 4.** The risk of T2D presence associated with fruit intake stratified by T2D-GRS tertiles in the subpopulations with no self-awareness of T2D or no dietary intervention for T2D.

**Supplemental Table 5.** The association of fruit intake with FPG, 2h-PG, HbA1c, Log-HOMA-β, and Log-HOMA-IR stratified by T2D-GRS tertiles.

**Supplemental Table 6.** Interactions of fruit consumption level with the rs10906115 and rs7172432on diabetes presence, FPG, 2h-PG, HbA1c, Log-HOMA-β, and Log-HOMA-IR.

**Supplemental Table 7.** The STROBE-nut recommendation checklist and the locations of relevant content in the present study.

**Supplemental Figure 1.** Flow chart for participant recruitment

**Supplemental Table 1.** Information and effect of each individual SNP adopted.

| **Gene** | **Chr.** | **Position** | **SNP** | **Effect Allele/other** | **EAF** | ***P* for**  **HWE** | **T2D** | | |
| --- | --- | --- | --- | --- | --- | --- | --- | --- | --- |
|  |  |  |  |  |  |  | **OR** | **95% CI** | ***P* value** |
| *PPARG* | 3 | 12351626 | rs1801282 | C/G | 0.94 | 0.0008 | 1.11 | 0.98, 1.26 | 0.11 |
| *KCNJ11* | 11 | 17387083 | rs5215 | C/T | 0.39 | 0.17 | 1.06 | 1.00, 1.13 | 0.06 |
| *TCF7L2* | 10 | 112998590 | rs7903146 | T/C | 0.03 | 0.10 | 1.23 | 1.05, 1.44 | 0.01 |
| *TCF2/HNF1B* | 17 | 37738049 | rs4430796 | G/A | 0.29 | 0.47 | 1.18 | 1.10, 1.26 | < 0.0001 |
| *IGF2BP2* | 3 | 185793899 | rs4402960 | T/G | 0.24 | 0.23 | 1.15 | 1.07, 1.23 | 0.0001 |
| *CDKN2A/B* | 9 | 22134095 | rs10811661 | T/C | 0.53 | 0.58 | 1.19 | 1.12, 1.26 | < 0.0001 |
| *HHEX/IDE* | 10 | 92703125 | rs1111875 | G/A | 0.27 | < 0.0001 | 1.06 | 0.99, 1.13 | 0.10 |
| *SLC30A8* | 8 | 117172544 | rs13266634 | C/T | 0.56 | < 0.0001 | 1.12 | 1.06, 1.19 | 0.0001 |
| *JAZF1* | 7 | 28140937 | rs864745 | T/C | 0.77 | 0.005 | 1.04 | 0.97, 1.11 | 0.31 |
| *CDC123/CAMK1D* | 10 | 12286011 | rs12779790 | G/A | 0.17 | 0.24 | 1.05 | 0.97, 1.14 | 0.22 |
| *IRS1* | 2 | 226229029 | rs2943641 | C/T | 0.94 | 0.22 | 1.33 | 1.17, 1.52 | < 0.0001 |
| *PROX1* | 1 | 213985913 | rs340874 | G/A | 0.38 | 0.65 | 1.04 | 0.98, 1.11 | 0.19 |
| GCKR | 2 | 27518370 | rs780094 | G/A | 0.47 | 0.85 | 1.02 | 0.96, 1.08 | 0.62 |
| *DGKB* | 7 | 15024684 | rs2191349 | T/G | 0.65 | 0.98 | 1.06 | 1.00, 1.13 | 0.06 |
| *BCL11* | 2 | 60357684 | rs243021 | A/G | 0.67 | 0.21 | 1.09 | 1.03, 1.17 | 0.007 |
| *CENTD2* | 11 | 72722053 | rs1552224 | A/C | 0.92 | < 0.0001 | 1.07 | 0.96, 1.19 | 0.24 |
| *KCNQ1* | 11 | 2670241 | rs231362 | C/T | 0.89 | 0.10 | 1.07 | 0.97, 1.17 | 0.18 |
| *TP53INP1* | 8 | 94948283 | rs896854 | A/G | 0.33 | 0.03 | 1.10 | 1.04, 1.17 | 0.002 |
| *KCNQ1* | 11 | 2818521 | rs2237892 | C/T | 0.67 | 0.47 | 1.22 | 1.14, 1.30 | < 0.0001 |
| *C2CD4A/C2CD4B* | 15 | 62104190 | rs7172432 | A/G | 0.61 | 0.27 | 1.06 | 0.99, 1.12 | 0.08 |
| *SPRY2* | 13 | 80143021 | rs1359790 | G/A | 0.72 | 0.12 | 1.11 | 1.04, 1.19 | 0.003 |
| *FITM2/R3HDML/HNF4A* | 20 | 44318326 | rs6017317 | G/T | 0.43 | 0.45 | 1.05 | 0.99, 1.11 | 0.13 |
| *UBE2E2* | 3 | 23294959 | rs7612463 | C/A | 0.79 | 0.78 | 1.14 | 1.05, 1.22 | 0.001 |
| *PTPRD* | 9 | 8879118 | rs17584499 | T/C | 0.09 | 0.006 | 0.96 | 0.87, 1.07 | 0.50 |
| *SRR* | 17 | 2312964 | rs391300 | G/A | 0.70 | 0.70 | 1.01 | 0.95, 1.08 | 0.75 |
| *CDC123/CAMK1D* | 10 | 12272998 | rs10906115 | A/G | 0.63 | 0.63 | 1.11 | 1.04, 1.18 | 0.001 |
| *PSMD6* | 3 | 64062621 | rs831571 | C/T | 0.64 | 0.03 | 1.00 | 0.94, 1.06 | 0.89 |
| *MAEA* | 4 | 1316113 | rs6815464 | C/G | 0.57 | 0.65 | 1.07 | 1.01, 1.14 | 0.03 |
| *ZFAND3* | 6 | 38139068 | rs9470794 | C/T | 0.29 | 0.20 | 1.05 | 0.98, 1.12 | 0.14 |
| *GCC1/PAX4* | 7 | 127524904 | rs6467136 | G/A | 0.88 | < 0.0001 | 0.93 | 0.86, 1.02 | 0.10 |
| *GLIS3* | 9 | 4287466 | rs7041847 | A/G | 0.45 | 0.80 | 0.99 | 0.93, 1.05 | 0.79 |
| *PEPD* | 19 | 33402102 | rs3786897 | A/G | 0.52 | 0.45 | 1.07 | 1.01, 1.14 | 0.10 |
| *CDKAL1* | 6 | 20685255 | rs35612982 | T/C | 0.40 | 0.38 | 1.23 | 1.16, 1.31 | < 0.0001 |
| *FTO* | 16 | 53779455 | rs9936385 | G/T | 0.12 | 0.54 | 1.12 | 1.02, 1.23 | 0.02 |

Odds ratio (OR), 95% confidence interval (CI) and *P* values were calculated from logistic regression model with present diabetes as the dependent viable and each SNP as the independent variable in an additive genetic model after adjustment for age, sex and body mass index. SNP: single nucleotide polymorphism; Chr.: chromosome; HWE: Hardy-Weinberg equilibrium; T2D, type 2 diabetes. The SNPs were showed in risk allele and risk allele frequency (EAF).

**Supplemental Table 2.** Demographic information, metabolic traits, and lifestyles categorized by fruit intake levels.

| Characteristics | < 1 times / week  (N = 2,061) | 1 - 3 times / week  (N = 3,075) | > 3 times / week  (N = 6,521) | *P* for Trend  (N=11,657) |
| --- | --- | --- | --- | --- |
| T2D-GRS | 34.86±3.88 | 34.54±3.95 | 34.48±3.87 | 0.0003† |
| Age, years | 64.00±9.84 | 64.20±9.92 | 62.47±9.63 | <0.0001† |
| BMI, kg/m^2^ | 25.54±3.59 | 25.24±3.53 | 25.15±3.47 | <0.0001† |
| SBP, mmHg | 139.51±19.96 | 137.49±20.66 | 135.86±20.21 | <0.0001† |
| DBP, mmHg | 78.14±10.52 | 77.03±10.41 | 77.03±10.07 | 0.0002† |
| Log-TG, mmol/L | 0.30±0.53 | 0.29±0.53 | 0.28±0.52 | 0.05 |
| TC, mmol/L | 5.04±1.18 | 4.80±1.20 | 4.99±1.16 | 0.39 |
| LDL-C, mmol/L | 2.92±0.90 | 2.81±0.90 | 2.91±0.88 | 0.14 |
| HDL-C, mmol/L | 1.21±0.33 | 1.17±0.33 | 1.23±0.33 | <0.0001† |
| FPG, mmol/L | 6.60±2.36 | 6.02±1.74 | 5.82±1.44 | <0.0001† |
| 2h-PG, mmol/L | 10.06±5.01 | 9.05±4.14 | 8.46±3.66 | <0.0001† |
| HbA1c, % | 6.27±1.42 | 6.09±1.09 | 5.89±0.88 | <0.0001† |
| Log-HOMA-β | 3.89±0.69 | 4.02±0.59 | 4.10±0.55 | <0.0001† |
| Log-HOMA-IR | 0.57±0.60 | 0.47±0.57 | 0.47±0.54 | <0.0001† |
| Male, n (%) | 950 (46.09) | 1179 (38.34) | 2021 (30.99) | <0.0001† |
| Smoking, n (%) | 451 (21.88) | 457 (14.86) | 740 (11.35) | <0.0001† |
| Drinking, n (%) | 296 (14.36) | 302 (9.82) | 439 (6.73) | <0.0001† |
| Vigorous PA, n (%) | 126 (6.11) | 127 (4.13) | 436 (6.69) | <0.0001† |
| Moderate PA, n (%) | 327 (15.87) | 448 (14.57) | 1190 (18.25) |  |
| Diabetes, n (%) | 837 (40.61) | 891 (28.98) | 1465 (22.47) | <0.0001† |
| Diabetes Awareness, n (%) | 574 (27.85) | 497 (16.16) | 640 (9.81) | <0.0001† |
| Exercise Intervention, n (%) | 460 (22.32) | 378 (12.29) | 545 (8.36) | <0.0001† |
| Diet Intervention, n (%) | 510 (24.75) | 455 (14.80) | 587 (9.00) | <0.0001† |
| Diabetes treatment, n (%) | 515 (24.99) | 423 (13.76) | 543 (8.33) | <0.0001† |

The *P* for trend were derived from the linear regression for continuous variables or the CMH Chi-square test for ordinary variables. T2D, type 2 diabetes; GRS, genetic risk score; BMI, body mass index; SBP, systolic blood pressure; DBP, diastolic blood pressure; Log-TG, log transformed triglyceride; TC, total cholesterol; LDL-C, low-density lipoprotein cholesterol; HDL-C, high-density lipoprotein cholesterol; FPG, fasting plasma glucose; 2h-PG, OGTT 2-hour plasma glucose; Log-HOMA-β, log transformed homeostasis model assessment for β cell function; Log-HOMA-IR, log transformed homeostasis model assessment for insulin resistance; PA, physical activity. *: *P* < 0.05; †: *P* < 0.01.

|  | Fruit Intake Frequency | | | | | | *P* for Interaction |
| --- | --- | --- | --- | --- | --- | --- | --- |
|  | < 1 times / week | | 1 - 3 times / week | | > 3 times / week | |  |
|  | OR (95% CI) | *P* value | OR (95% CI) | *P* value | OR (95% CI) | *P* value |  |
| No self-awareness of T2D  (N = 9,946) | 1.07  (1.03, 1.11) | 0.0005 | 1.06  (1.03, 1.10) | <0.0001 | 1.05  (1.03, 1.07) | <0.0001 | 0.34 |
| No dietary intervention for T2D  (N = 10,105) | 1.08  (1.04, 1.11) | <0.0001 | 1.07  (1.04, 1.10) | <0.0001 | 1.05  (1.03, 1.07) | <0.0001 | 0.10 |

**Supplemental Table 3.** The risk of T2D presence associated with T2D-GRS stratified by fruit intake in the subpopulations with no self-awareness of T2D or no dietary intervention for T2D.

Data are odds ratios (ORs) and 95% confidential intervals (CI). *P* values under each fruit intake level were derived from multiple logistic regressions using the fruit intake level and covariates as independent variables. *P* for interaction values were calculated using the T2D-GRS, fruit intake level, T2D-GRS × fruit intake level, and covariates together as independent variables. Analyses in both the two subpopulations adjusted for age, gender, body mass index, systolic and diastolic blood pressure, log transformed triglyceride, total cholesterol, low- and high-density lipoprotein cholesterol, smoking, drinking, physical activity and principal components of dietary factors. *: *P* < 0.05; †: *P* < 0.01.

|  | Fruit intake Levels | T2D-GRS | | | | | | *P* for Interaction |
| --- | --- | --- | --- | --- | --- | --- | --- | --- |
|  |  | Tertile 1 | | Tertile 2 | | Tertile 3 | |  |
|  |  | OR  (95% CI) | *P* value | OR  (95% CI) | *P* value | OR  (95% CI) | *P* value |  |
| No  self-awareness  of T2D  (N = 9,946) | < 1 times / week | 1.00 |  | 1.00 |  | 1.00 |  | 0.34 |
|  | 1 - 3 times / week | 0.79  (0.56, 1.12) | 0.47 | 0.74  (0.53, 1.02) | 0.09 | 0.82  (0.60, 1.13) | 0.64 |  |
|  | > 3 times / week | 0.76  (0.55, 1.04) | 0.17 | 0.82  (0.62, 1.11) | 0.71 | 0.76  (0.56, 1.02) | 0.11 |  |
| No dietary  intervention  for T2D  (N = 10,105) | < 1 times / week | 1.00 |  | 1.00 |  | 1.00 |  | 0.10 |
|  | 1 - 3 times / week | 0.49  (0.50, 0.95) | 0.18 | 0.70  (0.51, 0.94) | 0.07 | 0.68  (0.51, 0.91) | 0.26 |  |
|  | > 3 times / week | 0.66  (0.48, 0.89) | 0.04* | 0.74  (0.56, 1.00) | 0.25 | 0.59  (0.45, 0.78) | 0.002† |  |

**Supplemental Table 4.** The risk of T2D presence associated with fruit intake stratified by T2D-GRS tertiles in the subpopulations with no self-awareness of T2D or no dietary intervention for T2D.

Data are odds ratios (ORs) and 95% confidential intervals (CI). *P* values under each T2D-GRS tertiles were derived from multiple logistic regressions using the fruit intake level and covariates as independent variables. *P* for interaction values were calculated using the T2D-GRS, fruit intake level, T2D-GRS× fruit intake level, and covariates together as independent variables. Analyses in both the two subpopulations adjusted for age, gender, body mass index, systolic and diastolic blood pressure, log transformed triglyceride, total cholesterol, low- and high-density lipoprotein cholesterol, smoking, drinking, physical activity and principal components of dietary factors. *: *P* < 0.05; †: *P* < 0.01.

|  | Tertile 1 (n=3,885) | | Tertile 2 (n=3,885) | | Tertile 3 (n=3,887) | | *P* for Interaction |
| --- | --- | --- | --- | --- | --- | --- | --- |
|  | β±SE | *P* value | β±SE | *P* value | β±SE | *P* value |  |
| FPG, mmol/L |  |  |  |  |  |  |  |
| Model 1 | -0.21±0.03 | <0.0001† | -0.29±0.04 | <0.0001† | -0.44±0.04 | <0.0001† | <0.0001† |
| Model 2 | -0.22±0.04 | <0.0001† | -0.32±0.04 | <0.0001† | -0.47±0.04 | <0.0001† | <0.0001† |
| Model 3 | -0.05±0.03 | 0.01* | -0.06±0.03 | 0.07 | -0.18±0.04 | <0.0001† | 0.01* |
| 2h-PG, mmol/L |  |  |  |  |  |  |  |
| Model 1 | -0.42±0.08 | <0.0001† | -0.50±0.09 | <0.0001† | -0.90±0.09 | <0.0001† | <0.0001† |
| Model 2 | -0.48±0.08 | <0.0001† | -0.56±0.09 | <0.0001† | -1.00±0.09 | <0.0001† | <0.0001† |
| Model 3 | -0.08±0.07 | 0.25 | 0.03±0.08 | 0.68 | -0.32±0.08 | <0.0001† | 0.01* |
| HbA1c, % |  |  |  |  |  |  |  |
| Model 1 | -0.11±0.02 | <0.0001† | -0.16±0.02 | <0.0001† | -0.23±0.02 | <0.0001† | <0.0001† |
| Model 2 | -0.13±0.02 | <0.0001† | -0.19±0.02 | <0.0001† | -0.26±0.02 | <0.0001† | <0.0001† |
| Model 3 | -0.03±0.02 | 0.16 | -0.02±0.02 | 0.42 | -0.07±0.02 | 0.0004† | 0.03* |
| Log-HOMA-β |  |  |  |  |  |  |  |
| Model 1 | 0.07±0.01 | <0.0001† | 0.08±0.01 | <0.0001† | 0.11±0.01 | <0.0001† | 0.005† |
| Model 2 | 0.07±0.01 | <0.0001† | 0.09± 0.01 | <0.0001† | 0.11±0.01 | <0.0001† | 0.004† |
| Model 3 | 0.03±0.01 | 0.02* | 0.03±0.01 | 0.04* | 0.04±0.01 | 0.0003† | 0.24 |
| Log-HOMA-IR |  |  |  |  |  |  |  |
| Model 1 | -0.01±0.01 | 0.20 | -0.03±0.01 | 0.001† | -0.05±0.01 | <0.0001† | 0.02* |
| Model 2 | -0.02±0.01 | 0.14 | -0.04±0.01 | 0.0002† | -0.06±0.01 | <0.0001† | 0.01* |
| Model 3 | 0.01±0.01 | 0.44 | 0.002±0.01 | 0.88 | -0.02±0.01 | 0.05 | 0.21 |

**Supplemental Table 5.** The association of fruit intake with FPG, 2h-PG, HbA1c, Log-HOMA-β, and Log-HOMA-IR stratified by T2D-GRS tertiles.

The β-coefficients, standard error (SE), and *P* value under each tertile was derived from linear regressions model using the fruit intake level and covariates as independent variables; while the *P* for interaction were calculated using the T2D-GRS, fruit intake level, T2D-GRS × fruit intake level, and covariates together as independent variables. Model 1 adjusted for age, gender, and BMI; Model 2 additionally adjusted for SBP, DBP, Log-TG, TC, LDL-C, HDL-C, smoking, drinking, physical activity and principal components of dietary factors; Model 3 further adjusted for self-awareness of diabetes, exercise and diet intervention, and diabetic treatment. *: *P* < 0.05; †: *P* < 0.01.

**Supplemental Table 6.** Interactions of fruit consumption level with the rs10906115 and rs7172432on diabetes presence, FPG, 2h-PG, HbA1c, Log-HOMA-β, and Log-HOMA-IR.

|  | Diabetes | | FPG, mmol/L | | 2h-PG, mmol/L | | HbA1c, % | | Log-HOMA-β | | Log-HOMA-IR | |
| --- | --- | --- | --- | --- | --- | --- | --- | --- | --- | --- | --- | --- |
|  | OR (95% CI)  or β±SE | *P* value | β±SE | *P* value | β±SE | *P* value | β±SE | *P* value | β±SE | *P* value | β±SE | *P* value |
| rs10906115 | | | | | | | | | | | | |
| AA (39.99%) | 0.70  (0.60, 0.83) | <0.0001† | -0.18±0.03 | <0.0001† | -0.28±0.07 | <0.0001† | -0.07±0.02 | 0.0001† | 0.06±0.01 | <0.0001† | -0.01±0.01 | 0.21 |
| AG (45.79%) | 0.67  (0.61, 0.73) | <0.0001† | -0.07±0.03 | 0.02* | -0.04±0.06 | 0.50 | -0.03±0.02 | 0.07 | 0.02±0.01 | 0.03* | -0.003±0.01 | 0.76 |
| GG (14.21%) | 0.59  (0.54, 0.65) | <0.0001† | 0.05±0.05 | 0.31 | 0.11±0.11 | 0.34 | 0.02±0.03 | 0.46 | -0.01±0.02 | 0.67 | 0.02±0.02 | 0.21 |
| Interaction | -0.12±0.04 | 0.001† | -0.09±0.03 | 0.0002† | -0.24±0.06 | <0.0001† | -0.05±0.02 | 0.002† | 0.02±0.01 | 0.008† | -0.01±0.01 | 0.07 |
| rs7172432 | | | | | | | | | | | | |
| AA (37.44%) | 0.67  (0.55 0.77) | <0.0001† | -0.12±0.03 | 0.0001† | -0.18±0.07 | 0.01* | -0.06±0.02 | 0.0009† | 0.04±0.01 | 0.002† | -0.02±0.01 | 0.09 |
| AG (47.12%) | 0.66  (0.60, 0.72) | <0.0001† | -0.10±0.03 | 0.0005† | -0.14±0.06 | 0.03* | -0.03±0.02 | 0.10 | 0.03±0.01 | 0.005† | -0.003±0.01 | 0.72 |
| GG (15.44%) | 0.60  (0.54, 0.66) | <0.0001† | -0.05±0.05 | 0.30 | 0.08±0.11 | 0.47 | -0.03±0.03 | 0.34 | 0.04±0.02 | 0.02 | 0.03±0.02 | 0.10 |
| Interaction | -0.07±0.04 | 0.04* | -0.07±0.03 | 0.007† | -0.15±0.06 | 0.008† | -0.04±0.02 | 0.02* | 0.01±0.01 | 0.17 | -0.02±0.08 | 0.02* |

The odds ratio (OR, 95% confidential interval [CI]) and *P* value for diabetes presence were calculated by the logistic regression and the β-coefficients, SE, and *P* value for FPG, 2h-PG, HbA1c, Log-HOMA-β, and Log-HOMA-IR were calculated by the linear regression model. Statistics within each specific genotype were calculated with fruit intake level and covariates; while for the interaction, fruit intake level, SNP× fruit intake level, and covariates were evolved together as independent variables. Test for diabetes were times /formed under the Model 2 which adjusted for age, gender, body mass index, systolic and diastolic blood pressure, log-transformed triglyceride, total cholesterol, low- and high-density lipoprotein cholesterol, smoking, drinking, physical activity, and principal components of dietary factors; for FPG, 2h-PG, HbA1c, Log-HOMA-β, and Log-HOMA-IR, tests were times /formed under the Model 3 which further adjusted for self-awareness of diabetes, exercise and diet intervention, and diabetic treatment. *: *P* < 0.05; †: *P* < 0.01.

| **Supplemental Table 7. The STROBE-nut recommendation checklist and the locations of relevant content in the present study.** | | | |
| --- | --- | --- | --- |
| **Item** | Item Number | Recommendations | Locations of Relevant Contents in the Present Study |
| **Title and Abstract** | 1 | (a) Indicate the study’s design with a commonly used term in the title or the abstract. | The tilte; The first sentence in Methods of Abstract, Line 57-58 |
|  |  | (b) Provide in the abstract an informative and balanced summary of what was done and what was found. | Methods, Results, and Conclusion of Abstract, Line 57-74 |
|  |  | nut-1. State the dietary/nutritional assessment method(s) used in the title, abstract, or keywords. | The second sentence in Methods of Abstract, Line 58-60 |
| **Introduction** |  |  |  |
| Background Rationale | 2 | Explain the scientific background and rationale for the investigation being reported. | The first paragraph, Line 78-89;  The first two sentences of the second paragraph, Line 90-93, in Introduction |
| Objectives | 3 | State specific objectives, including any prespecified hypotheses. | The last two sentences of the second paragraph, Line 94-98, in Introduction |
| **Methods** |  |  |  |
| Study Design | 4 | Present key elements of study design early in the patimes /. | The first sentence on Study population, Line 101-104 |
| Settings | 5 | Describe the setting, locations, and relevant dates, including times /iods of recruitment, exposure, follow-up, and data collection. | Study population, Line 101-110; |
|  |  | nut-5. Describe any characteristics of the study settings that might affect the dietary intake or nutritional status of the participants, if applicable. | N.A. |
| Participants | 6 | Cross-sectional study—give the eligibility criteria and the sources and methods of selection of participants. | Study population, Line 101-110 |
|  |  | nut-6. Report particular dietary, physiological, or nutritional characteristics that were considered when selecting the target population. | No particular dietary or nutritional characteristics were considered when selecting the studied population. As part population of a national survey, the physiological traits were reported in the previous studies which was cited in the first paragraph of Study population. |
| Variables | 7 | Clearly define all outcomes, exposures, predictors, potential confounders, and effect modifiers. Give diagnostic criteria, if applicable. | Genetic loci selection and GRS construction, Line 114-127; Assessment of fruit intake frequency, Line 129-141;  Definition of diabetes, Line 143-148;  Assessment of covariates, Line 150-164; and in the Statistical analysis |
|  |  | nut-7.1. Clearly define foods, food groups, nutrients, or other food components. | The second sentence in Assessment of fruit intake frequency, Line 132-139 |
|  |  | nut-7.2. When using dietary patterns or indices, describe the methods to obtain them and their nutritional protimes /ties. | N.A. |
| Data Sources—Measurements | 8 | For each variable of interest, give sources of data and details of methods of assessment (measurement). Describe comparability of assessment methods if there is more than one group. | Genotyping and quality control, Line 114-117;  Assessment of fruit intake frequency, Line 129-139;  Assessment of covariates, Line 150-164 |
|  |  | nut-8.1. Describe the dietary assessment method(s), e.g., portion size estimation, number of days and items recorded, how it was developed and administered, and how quality was assured. Report if and how supplement intake was assessed. | The last two sentences in Assessment of fruit intake frequency, Line 132-141 |
|  |  | nut-8.2. Describe and justify food composition data used. Explain the procedure to match food composition with consumption data. Describe the use of conversion factors, if applicable. | N.A. |
|  |  | nut-8.3. Describe the nutrient requirements, recommendations, or dietary guidelines and the evaluation approach used to compare intake with the dietary reference values, if applicable. | N.A. |
|  |  | nut-8.4. When using nutritional biomarkers, additionally use the STROBE Extension for Molecular Epidemiology (STROBE-ME). Report the type of biomarkers used and their usefulness as dietary exposure markers. | N.A. |
|  |  | nut-8.5. Describe the assessment of nondietary data (e.g., nutritional status and influencing factors) and timing of the assessment of these variables in relation to dietary assessment. | N.A. |
|  |  | nut-8.6. Report on the validity of the dietary or nutritional assessment methods and any internal or external validation used in the study, if applicable. | The FFQ used was validated and adopted in our previously published study which was cited at the first sentence of Assessment of fruit intake frequency, Line 130-131 |
| Bias | 9 | Describe any efforts to address potential sources of bias. | The third, fourth, and the fifth paragraphs in Statistical analysis, Line 181-196 |
|  |  | nut-9. Report how bias in dietary or nutritional assessment was addressed, e.g., misreporting, changes in habits as a result of being measured, or data imputation from other sources. | The Assessment of fruit intake frequency, Line 129-139 |
| Study Size | 10 | Explain how the study size was arrived at. | The second paragraph in Study population, Line 106-110, and Supplemental Figure 1 |
| Quantitative  Variables | 11 | Explain how quantitative variables were handled in the analyses. If applicable, describe which groupings were chosen and why. | Statistical analysis, Line 184-188 |
|  |  | nut-11. Explain the categorization of dietary/nutritional data (e.g., use of N-tiles and handling of nonconsumers) and the choice of reference category, if applicable. | The last sentence in Assessment of fruit intake frequency, Line 139-141; and the second paragraph in Statistical analysis, Line 172-180 |
| Statistical Methods | 12 | (a) Describe all statistical methods, including those used to control for confounding. | Statistical analysis, Line 166-198 |
|  |  | (b) Describe any methods used to examine subgroups and interactions. | Statistical analysis, Line 172-188 |
|  |  | (c) Explain how missing data were addressed. | The second paragraph in Study population, Line 106-110 |
|  |  | (d) Cross-sectional study—if applicable, describe analytical methods taking account of sampling strategy. | Statistical analysis, Line 181-183 |
|  |  | (e) Describe any sensitivity analyses. | The third paragraph in Statistical analysis, Line 181-183 |
|  |  | nut-12.1. Describe any statistical method used to combine dietary or nutritional data, if applicable. | The first paragraph in Statistical analysis, Line 170-171 |
|  |  | nut-12.2. Describe and justify the method for energy adjustments, intake modeling, and use of weighting factors, if applicable. | N.A. |
|  |  | nut-12.3. Report any adjustments for measurement error, i.e., from a validity or calibration study. | N.A. |
| **Results** |  |  |  |
| Participants | 13 | (a) Report the numbers of individuals at each stage of the study. | The second paragraph of Study population, Line 106-110, and Supplemental Figure 1 |
|  |  | (b) Give reasons for nonparticipation at each stage. | The second paragraph of Study population, Line 106-110 |
|  |  | (c) Consider use of a flow diagram. | Supplemental Figure 1 |
|  |  | nut-13. Report the number of individuals excluded based on missing, incomplete, or implausible dietary/nutritional data. | The second paragraph of Study population, Line 106-110 |
| Descriptive Data | 14 | (a) Give characteristics of study participants (e.g., demographic, clinical, and social) and information on exposures and potential confounders. | The first paragraph of Results, Line 201-206 |
|  |  | (b) Indicate the number of participants with missing data for each variable of interest. | The second paragraph of Study population, Line 106-110;  The first paragraph of Results, Line 201-206;  Supplemental Table 2. Demographic information, metabolic traits, and lifestyles categorized by fruit intake levels. |
|  |  | nut-14. Give the distribution of participant characteristics across the exposure variables if applicable. Specify if food consumption of total population or consumers only were used to obtain results. | N.A. (For variables with large-enough sample size, distribution could be regarded as normal in statistical analyses. ) |
| Outcome Data | 15 | Cross-sectional study—report numbers of outcome events or summary measures. | The first paragraph of Results, Line 203 |
| Main Results | 16 | (a) Give unadjusted estimates and, if applicable, confounder-adjusted estimates and their precision (e.g., 95% confidence interval). Make clear which confounders were adjusted for and why they were included. | Since age, gender, and body mass index are the three confounders widely acknowledged in studies of diabetes, we give estimates adjusted for these three variables as the simplest model rather than unadjusted estimates. The other covariates were all common confounders in studies of diabetes. |
|  |  | (b) Report category boundaries when continuous variables were categorized. | The last sentence of Assessment of fruit intake frequency in Materials and methods, Line 139-141 |
|  |  | (c) If relevant, consider translating estimates of relative risk into absolute risk for a meaningful time times /iod. | N.A. |
|  |  | nut-16. Specify if nutrient intakes are reported with or without inclusion of dietary supplement intake, if applicable. | N.A. |
| Other Analyses | 17 | Report other analyses done—e.g., analyses of subgroups and interactions and sensitivity analyses. | Table 2, Table 3, Supplemental Table 5, and Supplemental Table 6 |
|  |  | nut-17. Report any sensitivity analysis (e.g., exclusion of misreporters or outliers) and data imputation, if applicable. | Supplemental Table 3 and Supplemental Table 4 |
| Key Results | 18 | Summarize key results with reference to study objectives. | The first paragraph of Discussion, Line 272-278 |
| Limitation | 19 | Discuss limitations of the study, taking into account sources of potential bias or imprecision. Discuss both direction and magnitude of any potential bias. | The sixth paragraph of Discussion, Line 317-323 |
|  |  | nut-19. Describe the main limitations of the data sources and assessment methods used and implications for the interpretation of the findings. | The sixth paragraph of Discussion, Line 323-331 |
| Interpretation | 20 | Give a cautious overall interpretation of results considering objectives, limitations, multiplicity of analyses, results from similar studies, and other relevant evidence. | The sixth paragraph of Discussion, Line 331-333 |
|  |  | nut-20. Report the nutritional relevance of the findings, given the complexity of diet or nutrition as an exposure. | The last paragraph of Discussion, Line 334-342 |
| Generalizability | 21 | Discuss the generalizability (external validity) of the study results. | The last two paragraph of Discussion, Line 323-332 and 339-342 |
| **Other Information** |  |  |  |
| Funding | 22 | Give the source of funding and the role of the funders for the present study and, if applicable, for the original study on which the present article is based. | Funding in Declaration |
| Ethics |  | nut-22.1. Describe the procedure for consent and study approval from ethics committee(s). | The last paragraph of Study population in Materials and methods |
| Supplementary Material |  | nut-22.2. Provide data collection tools and data as online material or explain how they can be accessed. | The last paragraph of Statistical analysis in Materials and methods, Line 111-112 |

**Supplemental Figure 1.** Flow chart for participant recruitment


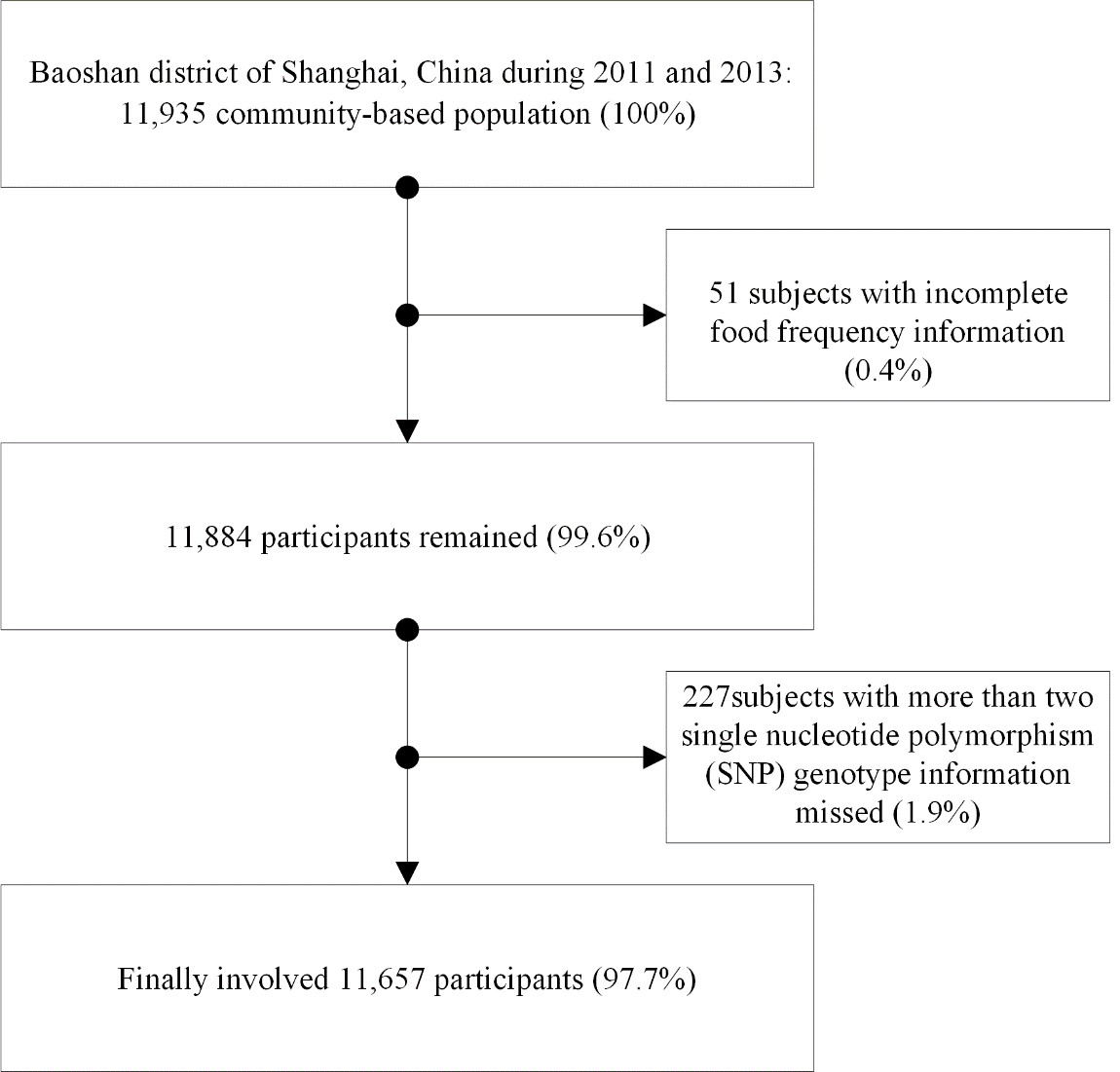

Supplement: Supplementary file 1 — Supplementary file1 (DOCX 123 KB) [file 394_2020_2449_MOESM1_ESM.docx]
